# Supplementary material for: Diacetyl Inhibits the Browning of Fresh-Cut Stem Lettuce by Regulating the Metabolism of Phenylpropane and Antioxidant Ability
Source: Foods. 2023 Feb 8;12(4):740. doi: 10.3390/foods12040740 (PMC9955407; doi:10.3390/foods12040740)
Supplement: Supplementary file 1 [file foods-12-00740-s001.zip › foods-2154652-supplementary.pdf]

Supplementary data:

Table S1 Primer sequence for real time-qPCR

Gene name	Forward primer sequences (5′-3′)	Reverse primer sequences (5′-3′)
**Actin**	AGCAACTGGGATGACATGGA	GGGTTGAGAGGTGCCTCAGT
**LsPAL1**	GGAAAACCGCAGAAGCAGTC	TTCTCGCAGAATCTCGACGG
**LsPAL2**	CGATAAGGTGTTCACTGCTTTG	TACACATTTGGCATGAGAGTTG
**LsPAL3**	GCTCGGAAGTGGGTTCTTAACA	CACACTCCAACAAGGGATCAAT
**LsPAL4**	AACAATGGCGAGAAGGAGAAA	TCGATCCTGTTAGGGATTGCTAA
**LsC4H**	CTTGAAGCACTCTACTGTCG	TCAAAATCTCCACATCACAC
**Ls4CL**	GTTTCTACACAAGTTGCTCC	TACAGTTTGGAGGTTGCAAG

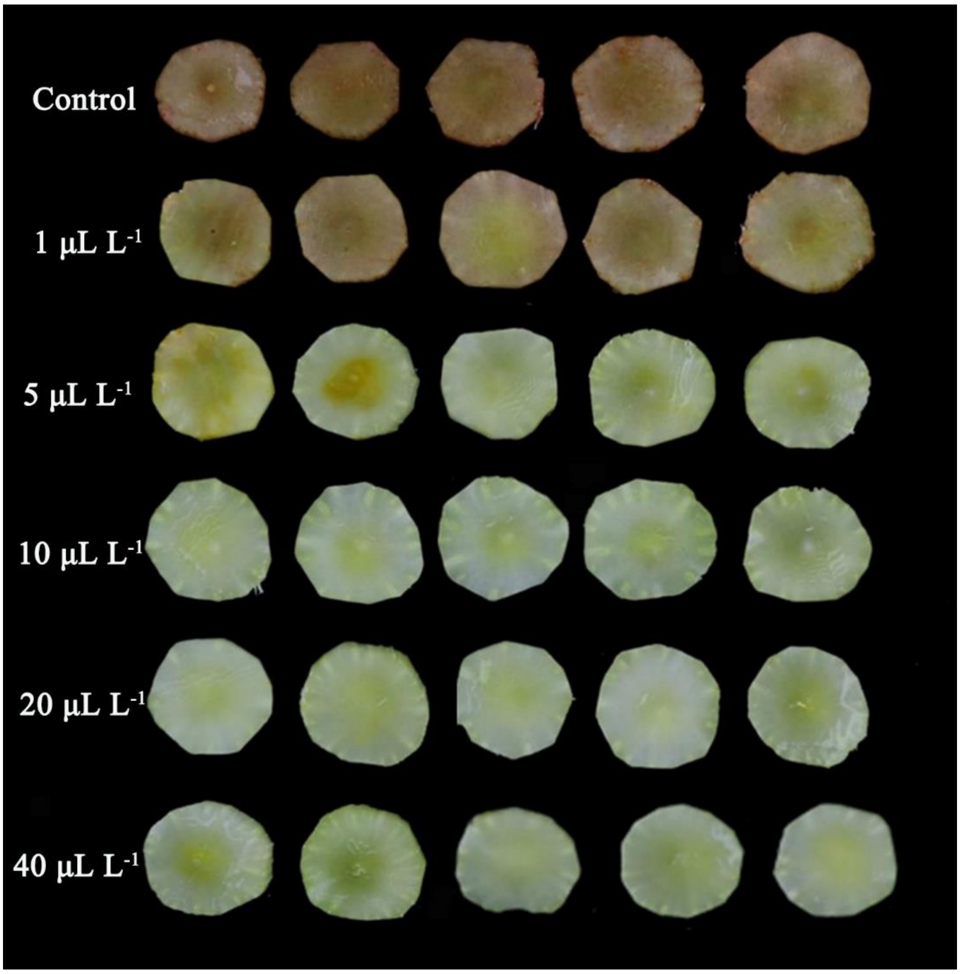

Figure S1. Appearance of fresh-cut stem lettuce treated with different concentrations of

diacetyl for 12 d at 4°C.

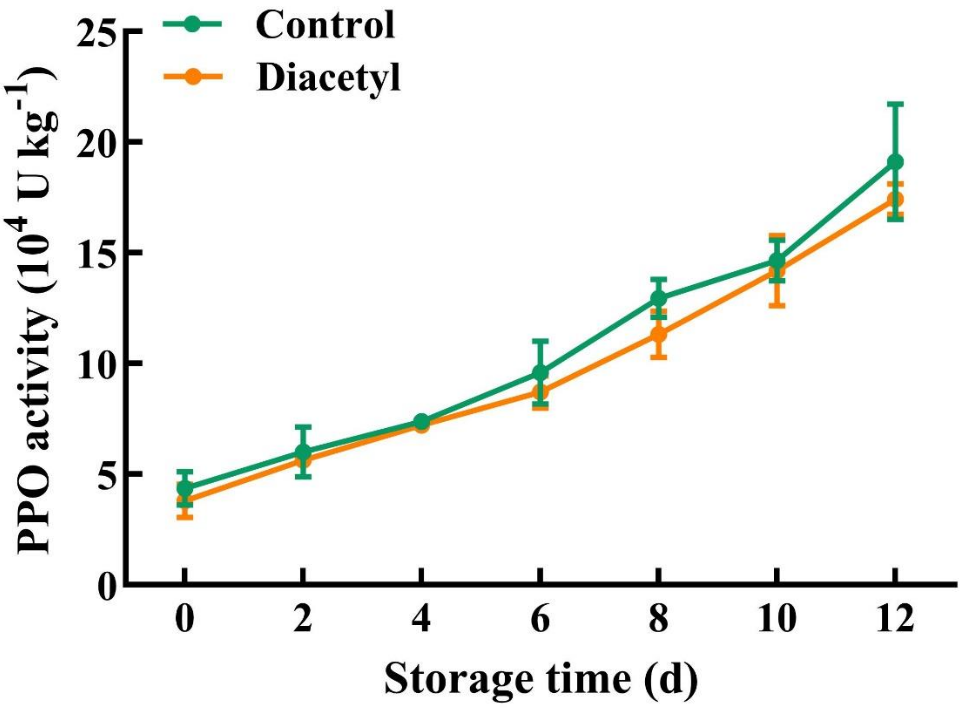

Figure S2. Effects of diacetyl treatment on PPO activity during storage time at 4 °C. Vertical bars represent standard deviations.
